# *In vivo* self-assembled albumin nanoparticle elicit antitumor immunity of PD-1 inhibitor by imaging and clearing tumor-associated macrophages

**DOI:** 10.3389/fchem.2024.1469568

**Published:** 2024-10-03

**Authors:** Cheng Yu, Linan Hu, Qilin Yu, Yulu Ren, Minping Zhang, Lujing Gao, Shiyi Lyu, Junli Wang, Enhua Xiao, Zhu Chen, Quanliang Shang, Pengfei Xu

**Affiliations:** ^1^ Department of Radiology, The Second Xiangya Hospital, Central South University, Changsha, Hunan, China; ^2^ Department of Radiology, Zhuzhou Central Hospital, Zhuzhou, Hunan, China; ^3^ Department of Ultrasound, The Air Force Hospital of Southern Theater Command, Guangzhou, Guangdong, China; ^4^ Department of Nuclear Medicine, Weifang People’s Hospital, Shandong Second Medical University, Weifang, Shandong, China

**Keywords:** albumin nanoparticle, tumor-associated macrophage, NIR-II imaging, CDT, immunotherapy

## Abstract

Eliciting anti-tumor immune responses and improving the tumor microenvironment crucial for boosting the effectiveness of anti-PD-1 immunotherapy. Tumor-associated macrophages (TAMs), the primary types of immune cells infiltrating tumors, play a critical role in the formation of an immunosuppressive microenvironment. In this study, we constructed a novel Evans Blue (EB)-based *in vivo* self-assembled nanocarrier system, mUNO-EB-ICG-Fc@Alb nanoparticles (designated as MA NPs), for targeted imaging and clearance of M2-TAMs to elicit antitumor immunotherapy of PD-1 inhibitor. *In vitro* experiments demonstrated the specific fluorescence imaging and killing effect of MA NPs on M2-TAMs. *In vivo* experiments shown that MA NPs-induced chemodynamic therapy (CDT) successfully reversed the tumor immunosuppressive microenvironment (ITM), promoted intratumoral infiltration of T lymphocytes, and ultimately enhancing the anti-tumor immunotherapy effect of PD-1 inhibitors. This study might provide good inspiration for improving the therapeutic efficacy of cancer immunotherapy.

## 1 Introduction

Immunotherapy has emerged as a viable and attractive treatment option for many cancer patients (Hargadon et al., 2018). Unlike previous surgical, chemotherapy, radiotherapy and targeted therapy, tumor immunotherapy is a therapeutic strategy that utilizes multiple means to stimulate and enhance the immune function of the body, and ultimately achieves the goal of removing tumor cells (Sanmamed and Chen, 2018). With the in-depth understanding of tumor immune escape mechanism, immune checkpoint inhibitors represented by Programmed Death 1/Programmed cell Death-Ligand 1(PD-1/PD-L1) inhibitors are becoming increasingly significant in cancer medication treatment (Li et al., 2021). Tumor tissues limit antitumor immunity by up-regulating immunosuppressive factors such as PD-1 ligand (PD-L1) that binds to PD-1 on tumor-specific CD8 T cells (Chen and Mellman, 2013). Drugs targeting the PD-1/PD-L1 immune checkpoint axis can block immunosuppressive signals and enable T cell–mediated elimination of cancer cells (Topalian et al., 2012). Monoclonal antibodies (mAbs) targeting the PD-1 have demonstrated impressive benefits for the treatment of some cancers (Zhan et al., 2016). However, PD-1 mAbs is not always effective, and we lack a complete understanding of the mechanisms that contribute to efficacy and resistance (Garon et al., 2015).

A key factor responsible for the poor response is the immunosuppressive tumor microenvironment (TME) mediated by tumor associated macrophages (TAMs) (Mathew et al., 2023; Yu et al., 2023). TAMs represent one of the main tumor-infiltrating immune cell types, play a crucial part in tumor invasion and metastasis (Quail and Joyce, 2013; Vitale et al., 2019). According to different activation signals, they are mainly classified as classical activated M1 macrophages and alternatively activated M2 macrophages (Hinshaw and Shevde, 2019). Usually, TAMs are dominated by M2 macrophages that promotes tumor progression in the tumor microenvironment (Yahaya et al., 2019; Fang et al., 2021). Mounting evidence indicates that TAMs are closely associated with non-response to anti-PD-1 therapy (Mantovani et al., 2017; Osipov et al., 2019; Zhao et al., 2019; Wang et al., 2023). The study by Gyo et al. found that PD-1 mAbs effectively bind PD-1 tumor-infiltrating CD8 T cells at early time points after administration. However, this engagement is transient, and PD-1 mAbs are captured and inactivated within minutes from the T cell surface by M2 macrophages (Arlauckas et al., 2017). These findings support M2 macrophages as a potential biomarker for treatment response or target to improve anti-PD-1 therapy in cancer. Although there are many studies dedicated to the repolarization of M2 macrophages to M1 macrophages, M1/M2 polarization is a dynamic equilibrium, and successfully polarized M1 macrophages may return to M2 macrophages under the influence of TME (Xu et al., 2022). Therefore, complete removal of M2-TAMs may be a more effective means (Zhu et al., 2021; Chen et al., 2023).

Photodynamic therapy (PDT) is an effective therapeutic modality that kills cells by utilizing photosensitizers to generate reactive oxygen species (ROS) under laser irradiation, with great spatiotemporal selectivity and minimal invasiveness (Dolmans et al., 2003; Xu et al., 2017). Indocyanine green (ICG) is the only near-infrared (NIR) dye approved for clinical application by the FDA (Hu et al., 2022). ICG has good optical properties and reactive oxygen generation ability, which can be used for near infrared second window (NIR-II) imaging and photodynamic therapy (Fang et al., 2019). However, the therapeutic benefits of PDT for deep tumors are greatly attenuated, due to the hypoxic tumor microenvironment and the attenuation of infrared light (Zhou et al., 2016; Dang et al., 2017). A promising strategy to amplify PDT effect is Fenton reaction-based chemodynamic therapy (CDT) (Liu et al., 2018). Catalyzed by Fe^2+^/Fe^3+^ ions, the cascade reaction can not only convert endogenous H2O2 into •OH, one of the most toxic ROS, but also produce O_2_ to improve the efficacy of PDT (Dai et al., 2018; Liu et al., 2019; Zhu et al., 2022). On the other hand, stable H_2_O_2_ is also produced during the PDT process, which promotes the virtuous circle of Fenton reaction (Li et al., 2020). Ferrocene (Fc) is an organometallic compound with reversible redox properties, which can achieve swift Fenton reaction in a physiological environment and provide ferrous ions to improve CDT efficacy (van Staveren and Metzler-Nolte, 2004; Wang et al., 2018). Therefore, constructing a nanoplatform to co-deliver Fc and ICG to tumor is a promising strategy for effective CDT (Chen et al., 2023; Ning et al., 2023).

**SCHEME 1:**
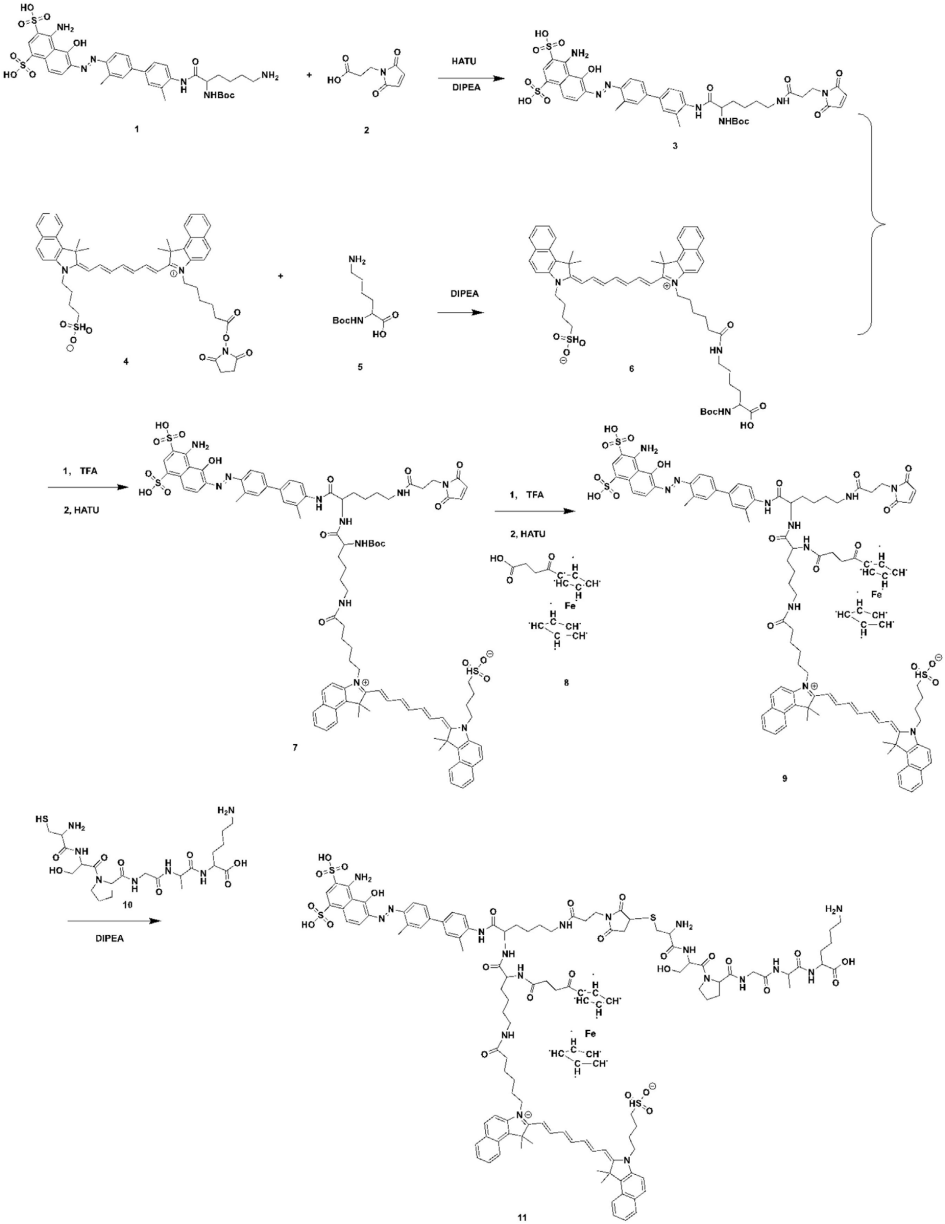
The synthesis of the mUNO-EB-ICG-Fc.

**SCHEME 2:**
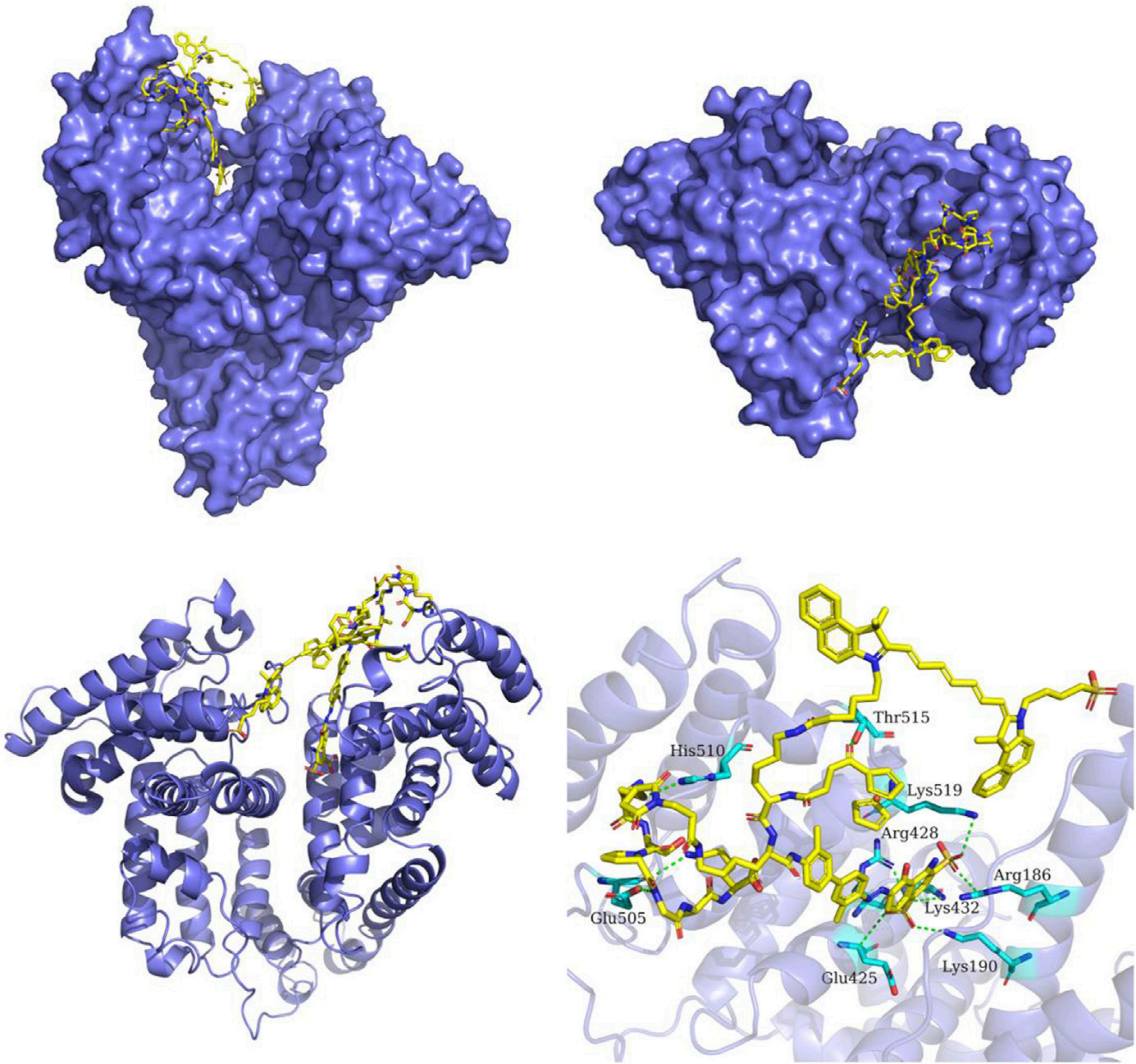
The predicted binding mode of MA NPs and serum albumin. The structure of serum albumin is colored in slate-blue. MA NPs is shown as yellow sticks. The interacting residues in serum albumin are shown as cyan sticks. The hydrogen bonds between MA NPs and serum albumin are depicted as green dashed lines.

Traditional nanocarriers can deliver drugs to the site of action, improve efficacy and reduce side effects, but themselves may cause toxic side effects and immunogenicity (Jacobson et al., 2016; Zhao et al., 2020). In this study, we constructed a novel Evans Blue (EB)-based drug self-delivery system (DSDS), mUNO-EB-ICG-Fc@Alb nanoparticles (designated as MA NPs), for targeted imaging and clearance of M2-TAMs to elicit antitumor immunotherapy of PD-1 inhibitor. The synthesis route is shown in Scheme 1. As a high-affinity albumin binding agent, EB can be assembled into nanoparticles with serum albumin *in vivo* and self-delivered to tumor areas (Ehlerding et al., 2018; Jaynes et al., 2020). The molecular docking model is shown in Scheme 2 mUNO is a short homing peptide that targets the marker of M2-macrophages, CD206 (Movahedi et al., 2010). After mUNO-mediated cell uptake, MA NPs generate fluorescence and ROS under laser excitation, achieving real-time quantitative NIR-II imaging and photodynamic effects on M2-TAMs. Moreover, the cascade Fenton reaction catalyzed by Fc generated •OH and O_2_ to relieve hypoxia and amplify PDT efficiency, achieving chemodynamic effects on M2-TAMs. In turn, ROS generated by PDT promoted shape-transformation and continuous occurrence of Fenton reaction, achieving complete clearance of M2-TAMs, thereby reversing the immunosuppressive microenvironment of tumors and eliciting the anti-tumor immunity of PD-1 mAbs. *In vitro* experiments have demonstrated the specific fluorescence imaging and killing effect of MA NPs on M2-TAMs. *In vivo* experiments shown that MA NPs-induced chemodynamic therapy (CDT) significantly improved the tumor immune microenvironment, promoted intratumoral infiltration of T lymphocytes, and ultimately enhancing the anti-tumor immunotherapy effect of PD-1 inhibitors. This study provides insights into potential therapeutic targets to improve the efficacy of immune checkpoint blockade and key clues to developing novel tumor immunotherapies.

## 2 Results and discussion

### 2.1 •OH generation performance of MA NPs

**FIGURE 1:**
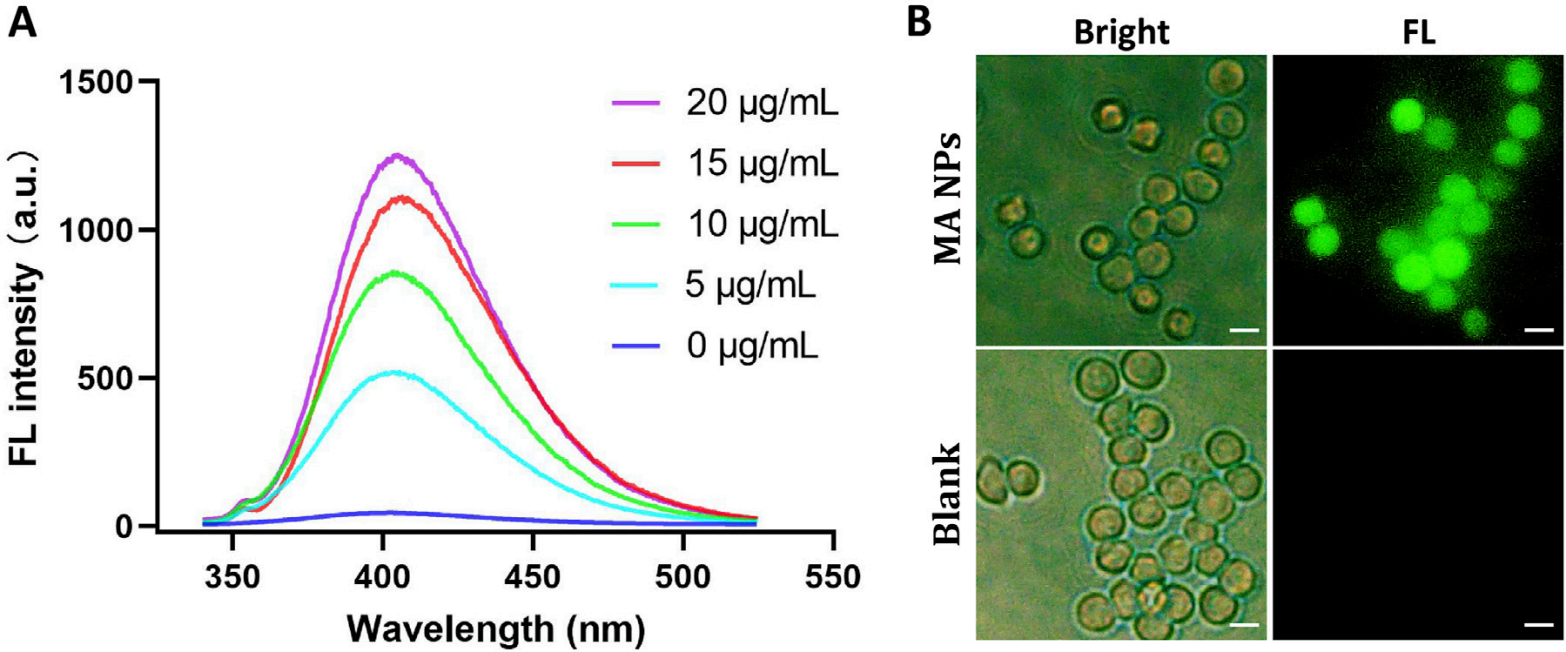
**(A).** FL emission spectra of the mixture of TA, H_2_O_2_, and MA NPs at different concentrations. **(B).** FL images of ROS in M2 macrophages treated with MA NPs with/without laser. Scale bar: 50 µm.

The efficient generation of highly toxic •OH is a crucial step in Fenton reaction-based CDT. Therefore, the catalytic ability of MA NPs for Fenton reaction was evaluated by terephthalic acid (TA), a fluorescence probe for detecting •OH. After TA was oxidized to 2-hydroxyp-benzoic acid by •OH, a characteristic fluorescence peak at 435 nm can be observed. As shown in Figure 1A, the fluorescence intensity of MA NPs group was much higher than that of the other control groups, revealing their promising ability in producing •OH. The concentration-dependent •OH generation behavior was also researched. The fluorescence intensity dramatically elevated with the increase of MA NPs concentration. 2′,7-dichlorodihydrofluorescein diacetate was used to further detect the •OH production ability of MA NPs at the cellular level to verify the feasibility of MA NPs as a CDT agent. As shown in Figure 1B, no obvious fluorescence was observed in MA NPs without laser irradiation group, indicating Fc hardly increased •OH level, due to ineffective Fenton reactions with insufficient endogenous H_2_O_2_ in M2 macrophages. In contrast, significant fluorescence intensity was observed in MA NPs with laser irradiation group, indicating the PDT effect of MA NPs could provide sufficient H_2_O_2_ for Fenton reactions to produce •OH. Overall, MA NPs can generate sufficient ROS within cells to achieve efficient CDT.

### 2.2 Cellular uptake study

**FIGURE 2:**
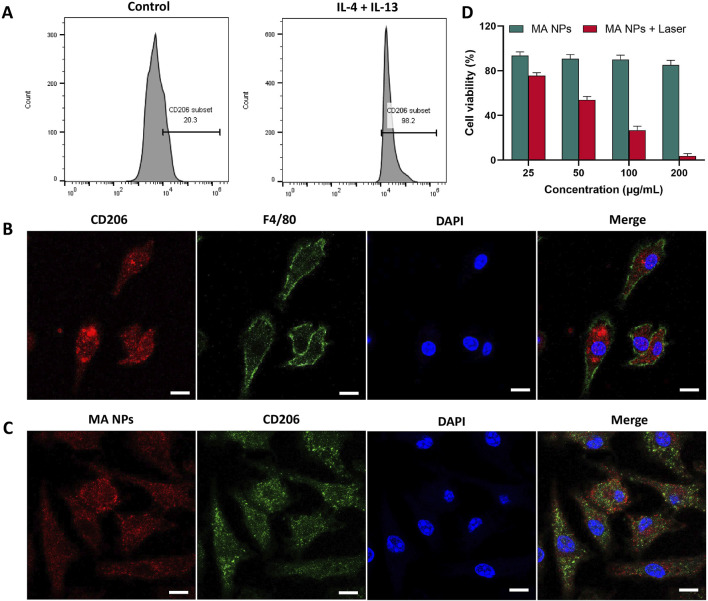
**(A)** Flow cytometry analysis and **(B)** immunocytochemical images showing BMDMs polarization to M2 functional phenotype using IL-4 and IL-13. **(C)** CLSM images of M2 macrophages treated with MA NPs. **(D)** Cell viability of M2 macrophages treated with MA NPs at various concentrations with and without laser irradiation. Scale bar: 20 µm.

The efficacy of MA NPs delivery to M2 macrophages was assessed via applying confocal laser scanning microscope (CLSM). We successfully extracted bone marrow cells and induced them to differentiate into M2 macrophages. Flow cytometry and immunofluorescence staining were used to confirm the markers of M2 macrophages. As shown in Figure 2A, a significant increase from 20.3% to 98.2% in the CD206^+^ M2 macrophages was induced after IL-4/IL-13 stimulation. Immunofluorescence co-staining of F4/80 and CD206 antibodies further determined the functional phenotype of M2 macrophages (Figure 2B). Subsequently, the cellular uptake of MA NPs on M2 macrophages was investigated by CLSM. As shown in Figure 2C, after incubation with MA NPs, the CLSM images displayed strong red fluorescence signals in the cytoplasm of M2 macrophages, which perfectly integrated with the green fluorescence signals of CD206 and blue fluorescence signals of DAPI. These results demonstrate that MA NPs could effectively target M2 macrophages for FL imaging and chemodynamic therapy.

### 2.3 *In vitro* CDT effects

The *in vitro* CDT Performance of Fc and MA NPs against M2 macrophages were evaluated by CCK-8 cell proliferation kit. In the MA NPs without laser irradiation group, negligible cytotoxicity was detected in M2 macrophages, even when the maximum concentration reached up to 200 μg/mL (Figure 4A). These results indicated that the conventional CDT efficacy with iron source supply only was severely compromised by insufficient endogenous H_2_O_2_ in M2 macrophages. Due to the fact that the PDT process has been proven to produce stable H_2_O_2_ for Fenton reaction to achieve efficient CDT effect, a significant killing effect of MA NPs on M2 macrophages can be observed under laser irradiation (Figure 2D). Meanwhile, the Fenton reaction can also generate O_2_ to enhance the PDT efficacy, which promotes the virtuous circle of CDT. Thus, only weak cell viability was observed at concentrations of MA up to 200 μg/mL. These results indicate that MA NPs could be utilized as an efficient nanoagents and achieve amplified CDT efficacy through self-supplying O_2_ and H_2_O_2_.

### 2.4 *In vivo* NIR-II imaging

**FIGURE 3:**
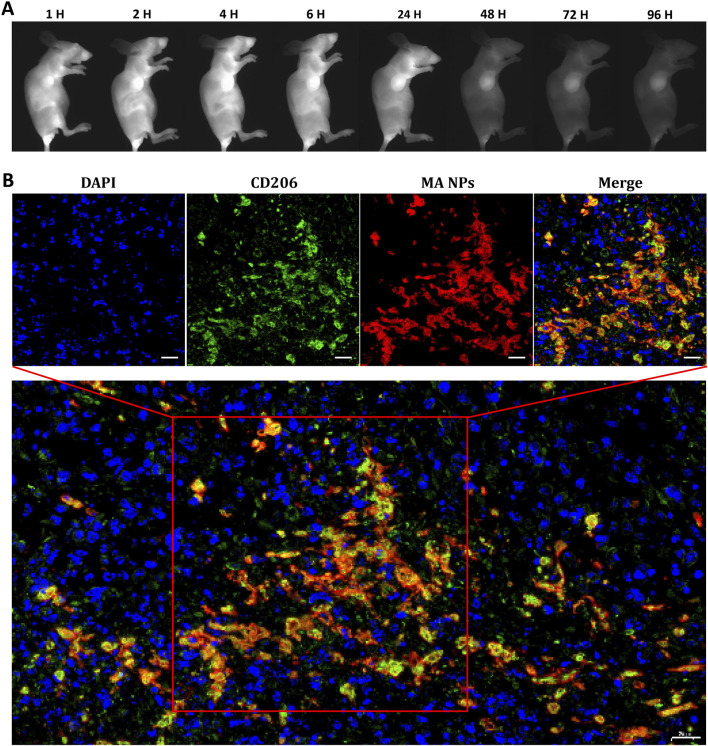
**(A)** *In vivo* NIR-II FL imaging of mice 4T1-bearing tumors. **(B)** MA NPs targets M2-TAMs in 4T1 tumor. Scale bar: 20 µm.

The *in vivo* targeted fluorescence imaging of MA NPs in TAMs was investigated in the 4T1 murine mammary carcinoma model, which is a typical immunosuppressive tumor model with abundant infiltrating macrophages (Movahedi et al., 2010; Gu et al., 2021). After tail vein injection of MA NPs, representative NIR-II fluorescence images at different time points were simultaneously recorded by NIR-II *in vivo* imaging system. As shown in Figure 3A, within 6 h after injection, MA NPs continues to accumulate in the tumor and reaches its peak, which was attributed to the EPR effect of tumors. Afterward, the fluorescence signal in the tumor gradually decreased, but the fluorescence signal in certain areas remained for 96 h. We speculate that the drugs in the tumor matrix are gradually cleared, while the drugs taken up by M2-TAMs remain in the tumor. Supplementary Figure S2 illustrates *ex vivo* fluorescence images of major organs and tumors harvested from mice at 96 h. There is almost no fluorescence in major organs, only the tumor has moderate fluorescence retention. Subsequently, the tumor tissue was stained for CD206, which is a marker of M2-TAMs. As shown in Figure 3B, the nearly coincident fluorescence of MA NPs and CD206 antibodies can be observed on confocal images, which demonstrates the targeting imaging ability of MA NPs for M2-TAMs in tumors.

### 2.5 *In vivo* therapeutic performance

**FIGURE 4:**
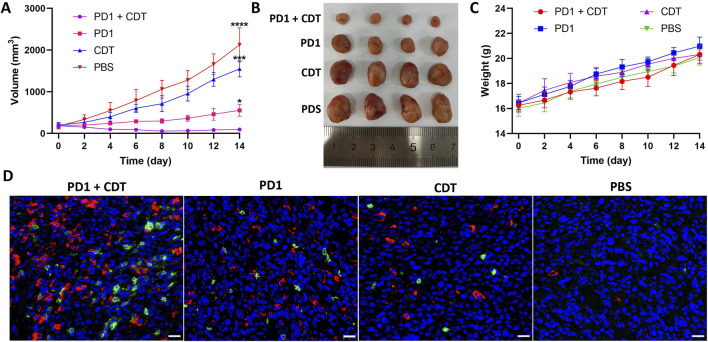
**(A)** Tumor volume evolvement curves of tumor-bearing mice. **(B)** Photographs of the dissected tumors by the end of treatment. **(C)** Body weight growth curve of tumor-bearing mice. **(D)** Immunofluorescence images for CD4 (red) and CD8 (green) of tumor sections. Scale bar: 20 µm.

Inspired by the satisfactory CDT efficacy *in vitro* and enhanced tumor accumulation *in vivo*, we further investigated the *in vivo* synergistic anti-tumor efficacy of MA *in vivo*. The 4T1 tumor-bearing BALB/c mice were randomly divided into 4 groups: (a) MA NPs + PD-1 mAbs + laser (b) PD-1 mAbs (c) MA NPs + laser (d) PBS. As shown in Figures 4A, B, compared to the control group, the tumors in the MA NPs + laser group exhibited slight restriction in tumor growth rate, indicating that the removal of M2-TAMs alone do not significantly affect tumor growth. Treatment with PD-1 mAbs alone inhibited limited growth of tumors, which was attributed to the immunosuppressive microenvironment in the tumor. However, the combination of M2-TAMs clearance and PD-1 mAbs treatment resulted in a maximal control of tumor burden and the smallest tumor volume, achieving the highest tumor inhibition ratio.

The recruitment of immune cells into the TME is a critical parameter directly associated with anti-tumor immune responses. Therefore, we used immunofluorescence staining to detect the infiltration of T lymphocytes in the tumor tissues of each group. As shown in Figure 4D, MA NPs + PD-1 mAbs + laser treatment showed a mass of CD4 (green) and CD8 T cells (red) infiltration in tumors, compared to the PD-1 mAbs group with limited tumor-infiltrating T cells. These results indicate that MA NPs can effectively clear M2-TAMs and eliminate the tumor immunosuppressive microenvironment, thereby significantly enhancing the anti-tumor immunotherapeutic effect of PD-1 mAbs. Moreover, no significant differences in body weight were observed in the corresponding groups, suggesting low systemic toxicity in all the treatments (Figure 4C).

## 3 Conclusion

In summary, we developed a *in vivo* self-assembled albumin nanoparticle MA NPs for eliciting antitumor immunity of PD-1 inhibitor. The precursor of nanoparticle was composed of mUNO for targeting M2-TAMs, ICG and ferrocene for NIR-II FL imaging guided CDT, and EB for albumin binding. When precursors enter the bloodstream, they quickly assemble into nanoparticles with serum albumin and self deliver to the tumor area. After mUNO-mediated cell uptake, MA NPs can effectively label and real-time fluorescence quantitative monitor M2-TAMs. The synergistic CDT of MA NPs significantly improved the immunosuppressive TME and elicited antitumor immunity of PD-1 inhibitor, which resulted in a large number of intratumoral infiltration of cytotoxic T lymphocytes and a significant tumor inhibition effect. This study might provide good inspiration for improving the therapeutic efficacy of cancer immunotherapy.

## Data Availability

The original contributions presented in the study are included in the article/Supplementary Material, further inquiries can be directed to the corresponding authors.
