# Supplementary material for: In vivo self-assembled albumin nanoparticle elicit antitumor immunity of PD-1 inhibitor by imaging and clearing tumor-associated macrophages
Source: Front Chem. 2024 Oct 3;12:1469568. doi: 10.3389/fchem.2024.1469568 (PMC11484263; doi:10.3389/fchem.2024.1469568)
Supplement: Supplementary file 1 [file DataSheet1.docx]

Supplementary Material

**In vivo self-assembled albumin nanoparticle elicit antitumor immunity of PD-1 inhibitor by imaging and clearing tumor-associated macrophages**

Cheng Yu^1^, Linan Hu^3^, Qilin Yu^1^, Yulu Ren^1^, Minping Zhang^1^, Lujing Gao^1^, Shiyi Lyu^1^, Junli Wang^4^,Enhua Xiao^1*^, Zhu Chen^1*^, Quanliang Shang^*^ and Pengfei Xu^2*^

**Experimental**

**Equipment and Methods**

Mouse M-CSF (315-02-10UG) Recombinant Protein was obtained from PeproTech (USA). Recombinant Murine IL-4 and IL-13 was obtained from PeproTech (USA). Anti-Mannose Receptor (CD206) antibody (ab64693) was obtained from Abcam (UK). F4/80 (BM8.1) Rat mAb (71299S) was obtained from CST (Cell Signaling Technology, USA). Fetal bovine serum (FBS) was obtained from Transgen (Invitrogen, USA). RPMI-1640 and Dulbecco’s modified Eagle’s medium (DMEM) medium was obtained from Gibco-Life Technologies (USA). Penicillin-streptomycin was obtained from Gibco-Life Technologies (USA).

**Preparation of mUNO-ICG-Fc-EB**

**Synthesis of Compound3.** Compound 1 was prepared according to the literature method with a slight modification. To a solution of compound 1 (154 mg, 0.2 mmol), N,N-Diisopropylethylamine (52 mg, 0.4 mmol) and O-(7-Azabenzotriazol-1-yl)-N,N,N',N'-tetramethyluronium hexafluorophosphate (76 mg, 0.2 mmol) in DMF (10 mL) was added compound 2 (35 mg, 0.2 mmol). The reaction mixture was stirred at room temperature for 2 h. DMF was removed under high vacuum and the residue was re-dissolved in methanol-H_2_O (1:1) and purified on a Interchim puriFlash 4250 system. MS analysis confirmed mass of 922.67 [M+H]^+^ with an isolated yield of 51% (94 mg).

**Synthesis of Compound 6.** Compound 4 (166 mg, 0.2 mmol) and compound 5 (50 mg, 0.2 mmol) were dissolved in DMF (10 mL) to which was added N,N-Diisopropylethylamine (52 mg, 0.4 mmol). The reaction mixture was stirred at room temperature for 12 hours. After the reaction was completed, the reaction mixture was concentrated in vacuo and the residue was purified by silica gel chromatography (DCM:MeOH = 20:1 v/v) to give Compound 6. MS analysis confirmed mass of 959.50 [M+H]^+^ with an isolated yield of 44% (84 mg).

**Scheme 1.** Synthetic route for compound 11

**Synthesis of Compound 7.** To a solution of compound 3 (92 mg, 0.1 mmol) in 4 mL DCM was added TFA (4 mL). The reaction was monitored by HPLC and it was completed in 2 hours. The reaction mixture was concentrated in vacuo and the residue was resolved in 5 mL DMF. Then, compound 6 (96 mg, 0.1mmol), N,N-Diisopropylethylamine (26 mg, 0.2mmol) and O-(7-Azabenzotriazol-1-yl)-N,N,N',N'-tetramethyluronium hexafluorophosphate (38 mg, 0.1 mmol) were added. The reaction was monitored by HPLC analysis and it was completed in 3hours. The mobile phases (A) demineralized water and (B) acetonitrile were acidified to pH 3 with trifluoroacetic acid. Gradient elution was performed as follows: 10% of B, 0-3 min;10-90% of B, 3-14 min; 90% of B, 14-16 min; 90-10% of B, 16-18min; 10% of B, 18-20min.The product was purified by Pro-HPLC.

**Synthesis of Compound 9**. The BOC groups of compound 7 (88 mg, 0.05 mmol) were removed using 30% TFA in DCM (v/v) for 2 hours at room temperature. After the TFA and DCM were removed by a flow of argon, compound 8 (14 mg, 0.05 mmol), O-(7-Azabenzotriazol-1-yl)-N,N,N',N'-tetramethyluronium hexafluorophosphate (19 mg, 0.05 mmol), N,N-Diisopropylethylamine (22 mg, 0.2 mmol) and DMF 5ml were added. The reaction was monitored by HPLC analysis and it was completed in 4 hours. The product was purified by Pro-HPLC. MS analysis confirmed mass of 1933.91 [M+H]^+^ with an isolated yield of 37 % (36 mg).

**Synthesis of Compound 11**. Compound 9 (19 mg, 0.01 mmol) and compound 10 (6 mg, 0.011 mmol) were dissolved in DMF (5 mL) to which was added N,N-Diisopropylethylamine (4.4 mg, 0.4 mmol). The reaction mixture was stirred at room temperature for 6 hours. The product was purified by Pro-HPLC. MS analysis confirmed mass of 1269.48 [M+2H]/2^+^ with an isolated yield of 32 % (8 mg).


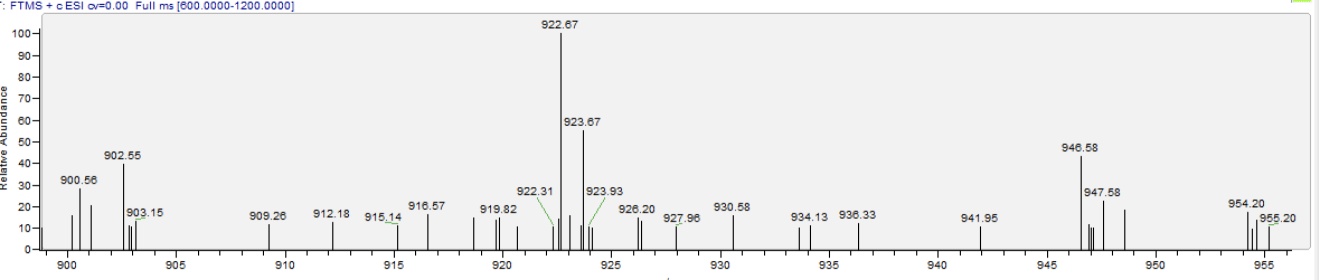


**Figure S1.** MS spectroscopy of compound 3.


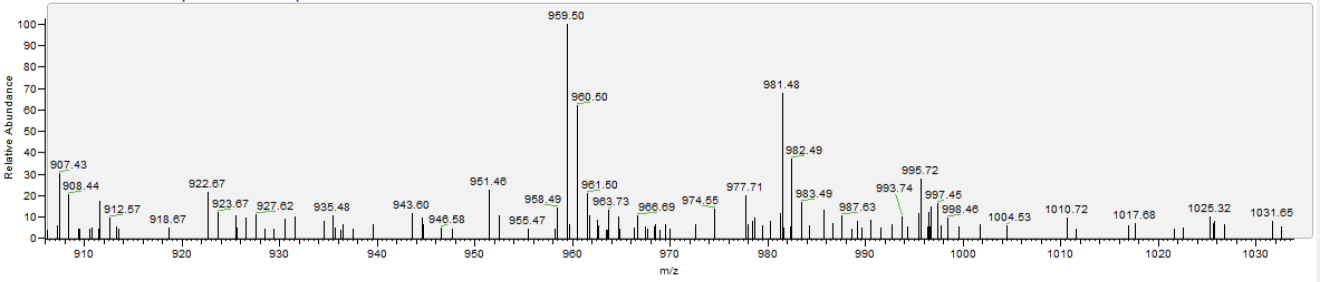


**Figure S2.** MS spectroscopy of compound 6.


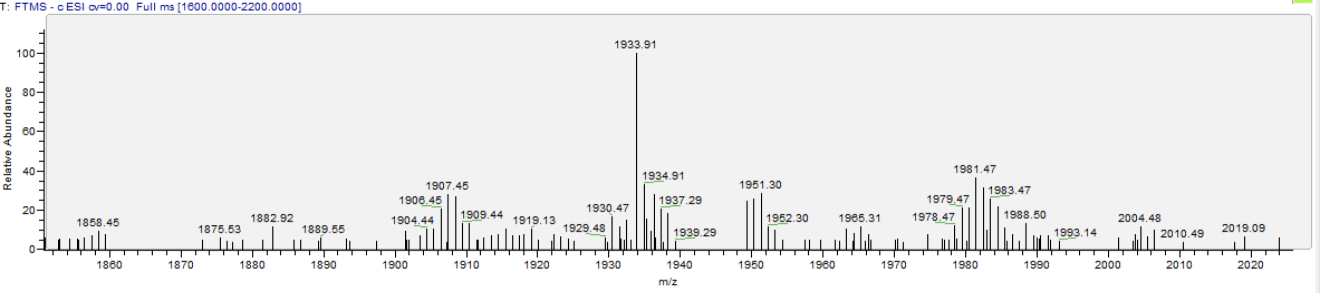


**Figure S3.** MS spectroscopy of compound 9.


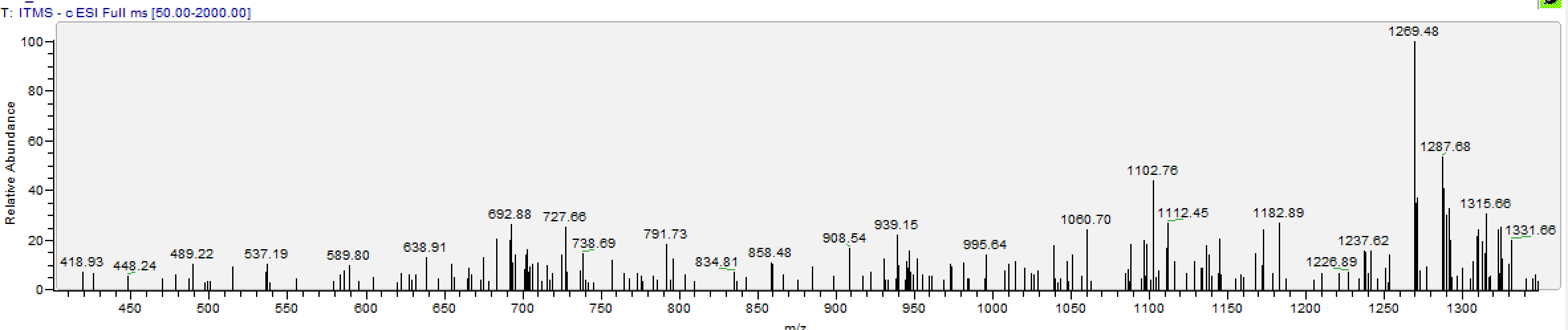


**Figure S4.** MS spectroscopy of compound 11.

**Cell Lines and Culture**

Mouse breast cancer 4T1 cells were obtained from Procell (Wuhan, China). M2 macrophages are obtained by inducing differentiation of bone marrow-derived macrophages (BMDMs) cultured in DMEM medium containing 10% fetal bovine serum. The extraction and induction of BMDMs are as follows. After anesthesia, 6-8 weeks old mice were dislocated and sacrificed to death. The sacrificed mice were then sterilized by soaking in 75% ethanol for 2 h. After disinfection, the femur and tibia of the mouse are dissected and placed inside a Petri dish containing 75% ethanol. After PBS washing, cut off both ends of the femur and tibia with scissors, and use a 1mL syringe to aspirate cold medium to blow the bone marrow out of the femur and tibia. The medium containing the bone marrow cells is pipetted repeatedly to disperse the cell clumps, and then the cells are sieved using a 70 μm cell filter. After centrifugation, discard the supernatant, add red blood cell lysate and resuspend for 5min. After centrifugation again, transfer the cells to a culture bottle containing 20 ng/mL M-CSF medium. M2 macrophages were generated by incubating the BMDMs in 20 ng/ml IL-4 and IL-13. Cells expressing F4/80 and CD206 are detected using fluorophore-conjugated antibodies to assess the formation of mature M2 macrophages. All cells used in this study were cultured in RPMI-1640 and DMEM medium supplemented with 10% FBS and 1% penicillin/streptomycin at 37 ℃ under 5% CO_2_.

**Detection of •OH**

The hydroxyl radicals were determined by fluorescence spectrometry with terephthalic acid (TA) as the probe. Different samples including i) MA NPs + TA + H_2_O_2_ and ii) TA + H_2_O_2_ were dispersed in phosphate buffer and kept in the incubator for 4 h. Concentration of H_2_O_2_ and TA were set as 1 mM and 0.5 mM, respectively, and the concentration of MA NPs were ranging from 5 to 20 µg/mL. The fluorescence spectra of these samples were then measured on a fluorescence spectrometer (λex= 315 nm). Intracellular •OH generation effect was investigated with DCFH-DA as a fluorescence probe. Adhered M2 macrophages were incubated with MA NPs (50 µg/mL) and PBS for 2 h, respectively. Then treated with DCFH-DA (10 µM) dispersed in serum-free culture medium for 4 h. All samples were washed with PBS before imaging via fluorescence microscope.

**CLSM images of cellular uptake**

The cellular uptake of MA NPs was investigated and imaged by a Zeiss LSM 710 confocal laser scanning microscopy (CLSM). Briefly, bone marrow cells were inoculated into confocal culture dishes and induced to differentiate into M2 macrophages. After incubation with MA (50 μg/ml) for 2 hours, cells were washed three times with PBS. Then, the mannose receptor were marked with CD206 antibody and the cell nuclei were marked with DAPI. The confocal fluorescence images were captured by CLSM.

**In vitro CDT effects**

The in vitro CDT effect was evaluated by a CCK-8 cell proliferation kit. M2 macrophages were seeded into a 96-well plate at a density of 5000 cells per well, and 5 replicate wells were set up. After overnight incubation, cells were cultured with MA NPs at different concentrations (12.5, 25, 50, 100, and 200 μg/mL) for 2 h. After cleaning with PBS and adding fresh culture medium, the experimental group was treated with laser irradiation (808nm, 0.5W/cm^2^) for 5 minutes, while the control group was not treated. Subsequently, 100 µL fresh medium containing 10% CCK-8 was added to each well. Finally, the 96-well plates were placed in a microplate reader (Thermo Fisher, USA) to measure the absorbance at 450nm.

**Tumor Model**

Female Balb/c mice (6-8weeks, average body weight of 18-20 g) were obtained from the Department of Experimental Animals of Central South University. All animal procedures were performed in accordance with the Guidelines for Care and Use of Laboratory Animals of Central South University and approved by the Animal Ethics Committee, The Second Xiangya Hospital, Central South University, China. To establish the tumor-bearing mouse model, 1 x 10^6^ 4T1 cells (100 μL) were subcutaneously injected into the right armpit of each mouse. Animal experiments were performed at around one week later.

**In Vivo FL Imaging**

In Vivo NIR-II Imaging and distribution of MA NPs were investigated on 4T1 tumor bearing mice. Firstly, MA solution was intravenously injected into the tail of the mice. After entering the bloodstream, MAs will quickly assemble with serum albumin to form MA NPs. Fluorescent pictures at different time points were captured using a NIR-II in vivo imaging system under inhalation anesthesia. At 96 h post-administration, the mice were immediately euthanized. The tumors and major organs of mice were excised to evaluate their fluorescence intensities.

**In vivo therapeutic effects**

To investigate the therapeutic performance of MA NPs in vivo, 4T1 tumor-bearing mice were randomly separated into four groups (n=4): (a) MA NPs (100 μL, 2 mg/mL) + PD-1 mAbs (100 μL, 2 mg/mL) + laser; (b) PD-1 mAbs (100 μL, 2 mg/mL); (c) MA NPs (100 μL, 2 mg/mL) + laser; (d) PBS (100 μL). The mice were treated on days 0 and 7. The prodrugs were injected into the bloodstream of mice through the tail vein and rapidly assemble into MA NPs with serum albumin. PD-1 mAbs were injected into the mouse body through intraperitoneal injection. NIR-II imaging was first performed prior to laser irradiation for outlining the tumor and further determining the range of the irradiation field. After 24 h post-injection, the tumor sites of mice were irradiated with 808 nm laser (1.0W/cm^2^, 10 min). Tumor volume and mice body weight were recorded periodically till day 14 post-treatment. The tumor volume (V) was calculated based on the following formula: V = (a × b^2^)/2, where a and b represent tumor length and width, respectively. After the experiment, the mice were euthanized and the tumor tissues were collected. The expression levels of CD4 and CD8 were detected by fluorophore-conjugated antibodies to evaluate the infiltration of T lymphocytes in tumor tissue.

**Statistical analysis**

All statistical analyses and graph generation were performed using GraphPad Prism 9 software. The statistical significance was determined at the level of p < 0.05.

**
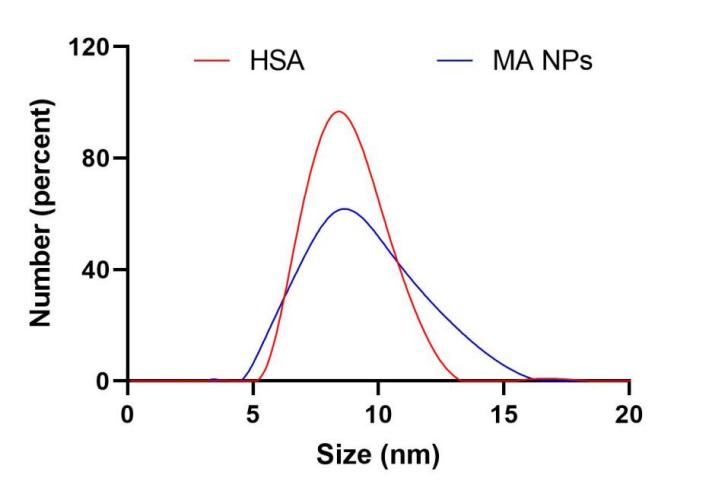
**

**Figure S5.** Hydrodynamic diameter

**
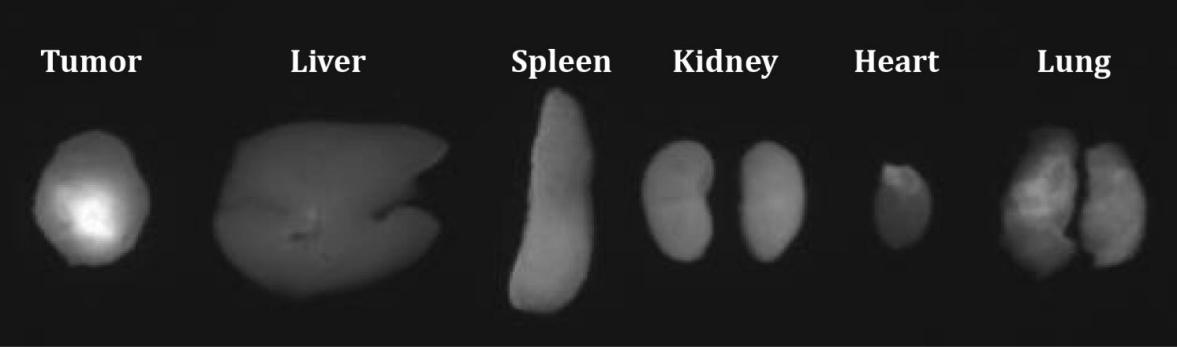
**

**Figure S6.** Ex vivo NIR-II fluorescence images of major organs and tumors.

**
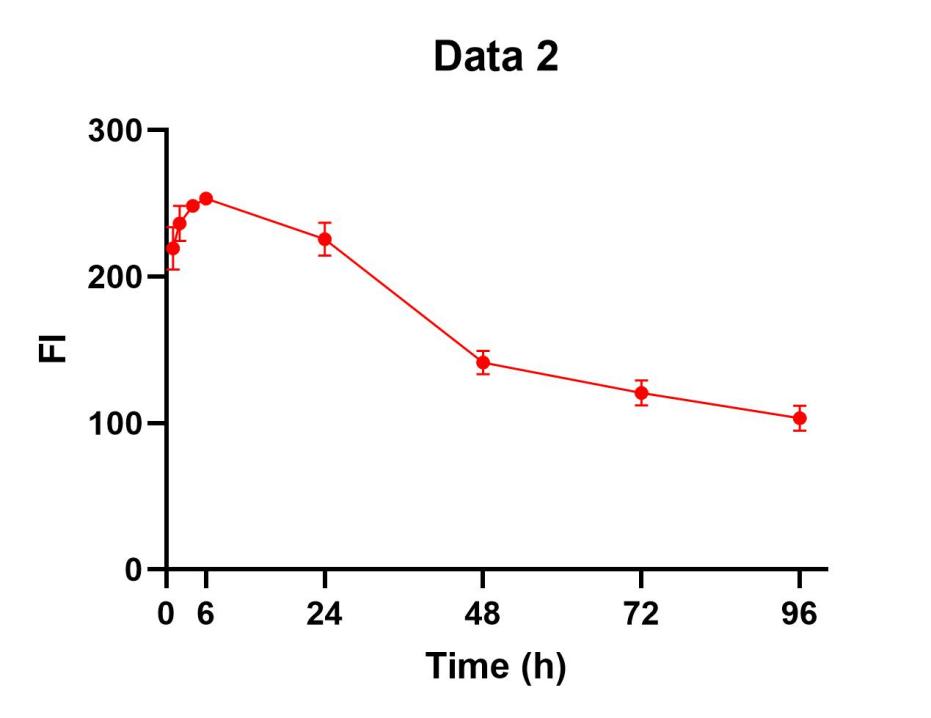
**

**Figure S6.** Quantitative analysis of tumor fluorescence intensity.
